# Monoclonal Antibodies as Therapeutic Agents in Autoimmune and Neurodegenerative Diseases of the Central Nervous System: Current Evidence on Molecular Mechanisms and Future Directions

**DOI:** 10.3390/ijms26199398

**Published:** 2025-09-26

**Authors:** Charalampos Skarlis, Efthalia Angelopoulou, Michail Rentzos, Sokratis G. Papageorgiou, Maria Anagnostouli

**Affiliations:** 1 Research Immunogenetics Laboratory, First Department of Neurology, School of Medicine, National and Kapodistrian University of Athens, NKUA, Aeginition University Hospital, Vas. Sofias 72-74, 11528 Athens, Greece; charskarlis@med.uoa.gr; 2 Multiple Sclerosis and Demyelinating Diseases Unit, Center of Expertise for Rare Demyelinating and Autoinflammatory Diseases of CNS, First Department of Neurology, School of Medicine, National and Kapodistrian University of Athens, NKUA, Aeginition University Hospital, Vas. Sofias 72-74, 11528 Athens, Greece; 3 Neurodegenerative Diseases and Rare Dementias Unit, First Department of Neurology, School of Medicine, National and Kapodistrian University of Athens, NKUA, Aeginition University Hospital, Vas. Sofias 72-74, 11528 Athens, Greece; angelthal@med.uoa.gr (E.A.); sokpapa@med.uoa.gr (S.G.P.); 4 Neuromuscular Diseases Unit, First Department of Neurology, School of Medicine, National and Kapodistrian University of Athens, NKUA, Aeginition University Hospital, Vas. Sofias 72-74, 11528 Athens, Greece; mrentzos@med.uoa.gr

**Keywords:** multiple sclerosis (MS), neuromyelitis optica spectrum disorder (NMOSD), Alzheimer’s disease (AD), Parkinson’s disease (PD), amyotrophic lateral sclerosis, monoclonal antibodies (mAbs), therapeutics, central nervous system (CNS), neuroinflammation, molecular mechanisms

## Abstract

Monoclonal antibodies (mAbs) have revolutionized the treatment landscape for neurological diseases, providing targeted, mechanism-based therapies for conditions ranging from autoimmune demyelinating disorders to neurodegenerative diseases. In multiple sclerosis (MS), mAbs against CD20, CD52, and α4-integrins offer disease-modifying efficacy by altering immune responses, depleting B cells, or blocking leukocyte migration into the central nervous system (CNS). Similarly, novel agents under investigation, such as frexalimab and foralumab, modulate T and B cell interactions and regulatory immunity. In neuromyelitis optica spectrum disorder (NMOSD), mAbs targeting IL-6, the complement cascade, and B cell lineage have demonstrated significant clinical benefit in preventing relapses and disability. In Alzheimer’s disease (AD), several anti-amyloid mAbs have gained regulatory approval. Anti-tau and anti-α-synuclein antibodies, though promising, have shown limited efficacy to date in AD and parkinson’s disease (PD), respectively. The evolving armamentarium of mAbs reflects a paradigm shift toward personalized neuroimmunology and neurodegeneration-targeted treatments, based on ongoing clarification of molecular and neuroinflammatory mechanisms. In this context, the present review summarizes current evidence on mAbs used in CNS disorders, with an emphasis on their pathophysiological targets, molecular mechanisms, clinical efficacy, and safety.

## 1. Introduction

Neurological diseases, including autoimmune, neurodegenerative, and demyelinating disorders, have historically posed significant therapeutic challenges due to their unclear etiology and complex pathophysiology.

Since the first approval of muromonab in 1985 [1], monoclonal antibodies (mAbs) have revolutionized therapeutics in neurology. By targeting specific antigens, mAbs offer selective therapies with constantly improving efficacy and safety. From pioneering murine-derived antibodies to modern humanized and fully human preparations, antibody engineering has minimized immunogenicity while maximizing therapeutic potential. With a growing number of ongoing clinical trials evaluating their effectiveness across the entire spectrum of neuroimmunology, the field is rapidly evolving.

Several mAbs initially approved for autoimmune neurological diseases have been repurposed for other indications, mainly hematological malignancies, as is the case for rituximab, alemtuzumab, and ofatumumab [2,3,4], and rheumatological conditions, as is the case for tocilizumab [5,6], while other mAbs, such as natalizumab, have been developed specifically for autoimmune neurological conditions, like multiple sclerosis (MS) [7]. Notably, mAbs are now expanding beyond traditional immune-mediated diseases, achieving significant breakthroughs in conditions such as Parkinson’s (PD) [8] and Alzheimer’s disease (AD) [9], conditions with evolving, growing evidence of neuroinflammatory components.

In the present review, we summarize the current knowledge regarding mAbs for the treatment of autoimmune demyelinating diseases like MS and neuromyelitis optica spectrum disorder (NMOSD) and neurodegenerative diseases (AD and PD) of the central nervous system (CNS). Given the rapidly growing evidence in the literature, we provide an updated overview of the emerging role of mAbs in the above-mentioned neurological disorders, focusing on their mechanisms, clinical advancements, and future directions, aiming to address current challenges.

## 2. Autoimmune Demyelinating Diseases of the CNS

### 2.1. Multiple Sclerosis

Multiple sclerosis (MS) is a chronic inflammatory demyelinating disease of the CNS, characterized by a complex interplay of autoimmune mechanisms resulting in myelin destruction, axonal damage, and progressive neurological disability. The main clinical phenotypes of the disease are relapsing–remitting MS (RRMS), which represents the most frequent disease form, and progressive MS, including secondary progressive (SPMS) and primary progressive (PPMS) forms. Both can be active or inactive, while the disease course is progressive over time [10,11]. The clinical manifestations of MS include vision disturbances such as unilateral visual loss or diplopia, weakness, dyscoordination, sensory loss or distortions, and changes in bowel and bladder function. Moreover, MS patients often complain of the so-called “hidden” symptoms, like cognitive impairment, fatigue, mood disturbances, and sleep disorders [11]. Although the etiology of MS remains unknown, several genetic, epigenetic, hormonal, and environmental factors have been linked with increased risk for disease development [12,13,14]. In this context, vitamin D deficiency, obesity, and smoking, active and passive, have been associated with an increased risk for MS [15,16,17,18]. Moreover, infectious agents such as the Epstein–Barr Virus (EBV) have been strongly associated with increased susceptibility to MS [19,20].

Gene variants related to antigen presentation and the modulation of immune responses have been associated with MS risk and/or pathogenesis [21,22,23], with the HLA-DRB1*15:01 allele being the strongest genetic risk for MS development, as has been shown in population and experimental autoimmune encephalomyelitis (EAE) studies [24,25,26,27,28]. Other HLA alleles, as well as non-HLA loci, were later found to be associated with a modest increase in MS risk [29]. Of note, recent studies suggest that the B-cell activating factor (BAFF) axis is associated with increased MS risk [30,31,32].

The disease pathophysiology involves a fundamental breach of immune tolerance, wherein autoreactive lymphocytes target myelin proteins, causing multifocal inflammation, demyelination, axonal destruction, and neurodegeneration. Initial disease pathogenesis begins when peripheral activated immune cells, particularly CD4^+^ and CD8^+^ T lymphocytes, B cells, and macrophages, traverse the compromised blood–brain barrier (BBB) and infiltrate the CNS. The transmigration of lymphocytes across the BBB is primarily mediated by the interaction between α4-integrin (also known as very late antigen-4, VLA-4) expressed on the surface of activated lymphocytes and vascular cell adhesion molecule-1 (VCAM-1) on endothelial cells. Within the CNS, these cells orchestrate a cascade of inflammatory processes: T cells release proinflammatory cytokines, activate microglia, and directly attack myelin and oligodendrocytes; B cells contribute through antigen presentation to T cells, cytokine secretion, and production of pathogenic autoantibodies against myelin components, while activated macrophages and microglia phagocytose myelin and release inflammatory mediators that further propagate tissue damage. This immune dysregulation results in the formation of demyelinating lesions (plaques) characterized by oligodendrocyte loss, myelin degradation, relative axonal preservation in early stages, and progressive axonal transection and neuronal death during the later phases [11,33,34,35,36].

**Figure 1:**
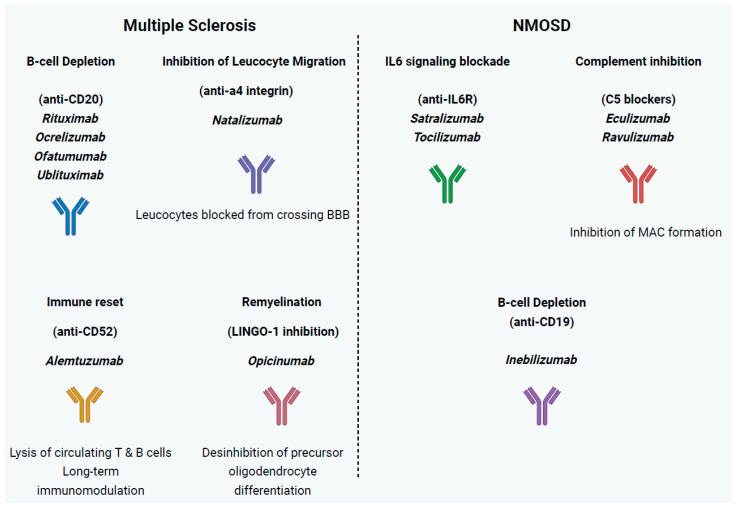
Mechanisms of action of monoclonal antibodies in multiple sclerosis (MS) and neuromyelitis optica spectrum disorders (NMOSDs). In MS, therapeutic strategies include (i) **B cell depletion** through anti-CD20 antibodies (rituximab, ocrelizumab, ofatumumab, ublituximab); (ii) **inhibition of leukocyte migration** via anti-α4 integrin therapy (natalizumab), which blocks immune cell entry across the blood–brain barrier; (iii) **immune reset** with anti-CD52 therapy (alemtuzumab), leading to lysis of circulating T and B lymphocytes and long-term immunomodulation; and (iv) **remyelination promotion** through LINGO-1 inhibition (opicinumab), enhancing oligodendrocyte precursor differentiation. In NMOSD, treatment approaches include (i) **IL-6 receptor blockade** (satralizumab, tocilizumab), dampening inflammatory signaling; (ii) **complement inhibition** at the C5 level (eculizumab, ravulizumab), preventing membrane attack complex (MAC) formation; and (iii) **B cell depletion** via anti-CD19 antibody (inebilizumab).

The elucidation of these cellular and molecular mechanisms has provided rational targets for mAb therapies. By selectively targeting key immune components such as CD20^+^ B cells (rituximab, ocrelizumab, ofatumumab, ublituximab), leukocyte adhesion molecules that facilitate BBB crossing (natalizumab), as well as T and B lymphocyte populations (alemtuzumab), mAbs have revolutionized MS treatment by selectively modulating disease-driving immune processes while preserving essential immune functions [3,37]. Furthermore, while classical disease-modifying therapies aim to prevent the formation of inflammatory lesions and slow disease progression, remyelination offers a promising alternative by restoring damaged white matter by promoting oligodendrocyte precursor cells (OPCs) differentiation into oligodendrocytes. Next, we describe mAbs according to the MS-related pathophysiological mechanism that they target (Figure 1 and Table 1).

#### 2.1.1. mAbs Inhibiting Leukocyte Migration into the CNS

Natalizumab is a humanized IgG4 mAb that has significantly advanced the treatment of RRMS by inhibiting the penetration of activated lymphocytes into the CNS. It is the first mAb specifically designed for MS; it binds to the α4 subunit of α4β1-integrin (CD49d) expressed on the surface of lymphocytes, thereby inhibiting their interaction with VCAM-1 on endothelial cells of the BBB. It was the first approved mAb for MS in 2004, showing superior efficacy compared to placebo [47] and better effectiveness when combined with interferon-β-1a compared to interferon-β-1a monotherapy [48,49]. Moreover, it has been routinely used worldwide in the treatment of pediatric-onset MS patients (POMS) with high disease activity [50,51,52]. Of importance, compared to all other agents, the use of natalizumab is associated with the highest risk of progressive multifocal leukoencephalopathy (PML), a rare but potentially fatal opportunistic infection linked to John Cunningham (JC) virus reactivation, necessitating careful patient selection [42]. This risk led to the transient withdrawal of its use for some months before it was FDA-reapproved in 2006 [53]. The introduction of the JC virus (JCV) index and an associated monitoring program enables appropriate risk stratification according to antibody titer levels, treatment duration, and immunosuppression history [42]. The risk of developing PML varies, from approximately 1 in 10,000 among patients with low JCV titers and no prior immunosuppression to as high as 1 in 70 in those with elevated titers and a history of immunosuppressive therapy [42]. Despite the severity of PML, reliable predictive markers are rather limited. In this context, recent studies revealed the effect of gene variants in immunosuppressing drug-exposed PML patients with complement component 8 (C8B) rs139498867, lymphocyte antigen 9 (LY9) rs763811636, Ficolin 2 (FCN2) rs76267164, and syntaxin binding protein 2 (STXBP2) rs35490401 variants, who were associated with a higher risk for PML compared to drug-exposed matched controls [67]. Moreover, the BAFF TTT haplotype derived from the rs9514828, rs1041569, and rs9514827 variants was found to confer significantly higher risk for anti-JCV seropositivity and indirectly for PML among male MS patients [68]. Other adverse events could be present according to the natalizumab recipient’s HLA immunogenetic background. Specifically, it was found that HLA-DRB1*13 and HLA-DRB1*14 alleles were significantly increased in patients who developed anaphylactic/anaphylactoid reactions to natalizumab therapy [69]. Additionally, it was found that a single-nucleotide polymorphism in the MerTK gene is associated with increased radiological disease activity in patients with multiple sclerosis on natalizumab therapy [70].

Despite this, natalizumab remains a highly effective option for patients with RRMS, particularly when other disease-modifying therapies have failed or are not tolerated.

#### 2.1.2. B Cell Depletion mAb Therapies

B-cell depletion therapy with anti-CD20 mAbs has emerged as a cornerstone of disease-modifying treatment for MS, by robustly targeting B cells in the CNS and periphery. In the periphery, B cell populations are controlled by regulatory T cells (Tregs) and CD8^+^ T cells within germinal centers, differentiating into pathogenic memory B cells via interactions with follicular Th cells. These pathogenic B cell subsets, expressing chemokine receptors CXCR3 and CCR6 alongside proinflammatory cytokines and adhesion molecule VLA-4, infiltrate the CNS across the BBB to encounter T cells within follicle-like structures, driving clonal expansion. Once inside the CNS, memory B cells mature into plasmablasts that secrete autoantibodies and proinflammatory cytokines, which further activate astrocytes, microglia, and CD4^+^ T cells, thereby contributing to inflammation and disease progression [3,24,70,71].

Rituximab, a chimeric IgG1κ antibody, binds to amino acid residues 168–176 on the large extracellular loop of the CD20 protein expressed on B cells, triggering complement-dependent cytotoxicity (CDC) and antibody-dependent cell-mediated cytotoxicity (ADCC), leading to B cell depletion. Although rituximab is widely used in clinical practice and approved for other indications, its use in neurological diseases remains off-label. Nevertheless, substantial clinical data support its long-term safety and effectiveness in MS, demonstrating favorable outcomes both when used as an initial treatment compared to approved therapies and when employed as switch therapy following natalizumab discontinuation, compared to fingolimod [39,40,41].

Despite the failure of a clinical trial of rituximab in primary progressive MS (PPMS) [43], further studies led to the development of ocrelizumab, the first effective treatment in PPMS that was approved in 2017 [44,45,53]. Ocrelizumab, a recombinant humanized IgG1κ mAb, targets the large extracellular loop of CD20, primarily inducing B cell apoptosis via ADCC. In a clinical trial involving patients with PPMS, treatment with ocrelizumab resulted in a 24% relative risk reduction in disability progression over 12 weeks compared to placebo [44]. While progressive multifocal leukoencephalopathy (PML) remains a potential risk, reported cases only refer to transitions from fingolimod or natalizumab [46].

Ofatumumab, a fully human IgG1κ antibody, binds to discontinuous sequences on both the small and large extracellular loops of CD20, resulting in potent CDC and ADCC-mediated B cell depletion, and is administered subcutaneously [72]. In a phase II trial in RRMS, ofatumumab reduced enhancing lesions at 12 weeks across multiple dosing regimens, emphasizing the unclear optimal extent of B-cell suppression for these patients [72]. It was approved for RRMS in 2021 [73].

Ublituximab is another B-cell depletion therapy approved for RRMS, and it is administered intravenously. It is a chimeric IgG1κ anti-CD20 mAb with a glycoengineered Fc segment, enhances affinity for FcγRIIIa receptors, thereby maximizing ADCC-mediated B cell depletion through binding to residues 168–171 and 158–159 on the large extracellular loop of CD20. In two phase III trials in RRMS, ublituximab reduced annual relapse rates and radiographic MRI lesions compared to teriflunomide, but it was not superior in terms of disability worsening [74]. However, post hoc analyses demonstrated that ublituximab resulted in no evidence of disease activity (NEDA) in more patients during certain periods compared to teriflunomide [54], suggesting a time-dependent benefit that needs to be further explored.

The variations in epitope binding, effector mechanisms, and degree of humanization contribute to differences in immunogenicity, dosing schedules, and pharmacokinetic profiles among the anti-CD20 mAbs used in MS.

Pre-treatment testing and safety monitoring are paramount when using anti-CD20 mAb therapy, with contraindications including active hepatitis B infection and a history of life-threatening infusion reactions. Recommended pre-treatment screening involves a complete blood count, a comprehensive metabolic panel, Quantiferon-tuberculosis screening, hepatitis panel, varicella-zoster virus (VZV) IgG, quantitative Ig levels, urine or serum beta-human chorionic gonadotropin (where appropriate), and brain MRI [55,56]. Post-treatment monitoring should include complete blood counts every month, comprehensive metabolic panels, CD19 counts every 6 months, quantitative Ig levels for recurrent or serious infections, monitoring for malignancy, and pregnancy/family planning considerations. Given that inflammatory disease activity can occur despite the lack of evidence for B-cell reconstitution based on CD19^+^ B cell counts, monitoring specific B cell subsets, particularly memory B cells, might provide a more predictive biomarker for disease activity [71].

Newly established innovative methodology has identified potential soluble markers for monitoring of ocrelizumab treatment in MS, combining immunophenotype findings, serum proteomics, serum neurofilament light chain measurements, etc. [57]. The future aim is to incorporate all these methods into everyday clinical neurological practice.

#### 2.1.3. mAb Immune Reset

Alemtuzumab is a humanized IgG1 mAb directed against CD52, an antigen expressed on the cell surface of mature lymphocytes. Alemtuzumab induces the depletion of circulating B and T cells, followed by a gradual, long-lasting repopulation process. Unlike many other immunotherapies, alemtuzumab is administered intravenously at a dose of 12 mg daily during two brief annual courses [53], with additional infusions only in recurrent disease activity. In two phase III trials, alemtuzumab demonstrated superior efficacy in reducing relapse rates in treatment-naïve patients and in those with inadequate prior response to interferon-β-1a or glatiramer acetate [58]. Five-year follow-up confirmed persistent suppression of clinical and radiologic disease activity, with approximately two-thirds of patients not requiring additional alemtuzumab infusions beyond the initial two sessions. Importantly, a notable reduction in brain volume loss was also observed [75,76].

Despite its robust clinical efficacy, alemtuzumab has been associated with an increased risk for infusion-related reactions, herpetic and other serious infections, and secondary autoimmune disorders, peaking in the third year post-treatment [77].

Open-label follow-up demonstrated that secondary autoimmune thyroid disorders affected over 40% of patients [78], with new cases of immune thrombocytopenic purpura (ITP) and antiglomerular basement membrane disease also reported [75,76]. Postmarketing surveillance data highlighted the importance of prophylactic antiviral treatment (e.g., Acyclovir) and also brought attention to Listeria meningitis cases, primarily within the first month after infusion, prompting formal recommendations for Listeria prophylaxis, especially in the United Kingdom [59]. Rarer adverse effects include such as acalculous cholecystitis [60], hemophagocytic lymphohistiocytosis [79], and acute coronary syndrome during infusion [80]. Alemtuzumab was approved for MS in 2014, but in 2019, its use was restricted by the EMA because of life-threatening side effects, until new data were available [53].

#### 2.1.4. mAbs Promoting Remyelination

Opicinumab is a fully human IgG1 mAb targeting leucine-rich repeat and immunoglobulin domain-containing neurite outgrowth inhibitor receptor-interacting protein-1 (LINGO-1), a known inhibitor of oligodendrocyte precursor cell (OPC) differentiation into mature oligodendrocytes [81]. In a phase II study in patients with acute optic neuritis, six-month treatment with opicinumab was well-tolerated but ineffective [81]. In another phase II trial in RRMS, opicinumab failed to achieve the clinical primary outcome [61]. Given these disappointing results, as of now, opicinumab is no longer being pursued as a treatment for MS.

The mAb rHIgM22, which was derived from a patient with Waldenström’s macroglobulinemia and promotes remyelination in animal models, has also been studied in MS [62]. Although the precise mechanism of action remains unclear, data derived from phase I trials indicate that it is well-tolerated and capable of limited BBB penetration [62]. Further clinical trials are required to evaluate CNS bioavailability and therapeutic potential in humans.

#### 2.1.5. Other mAbs in MS

Daclizumab, a humanized IgG1 mAb targeting IL-2 receptor alpha (CD25), was initially developed for the prevention of kidney transplant rejection, before being adapted for RRMS in the form of daclizumab via a high-yield process [63]. Despite its clinical efficacy, its use was limited due to serious safety concerns, particularly autoimmune hepatitis [63]. During postmarketing surveillance, additional autoimmune complications were reported, including twelve cases of severe inflammatory CNS disorders that resulted in at least three deaths. As a result, daclizumab was voluntarily withdrawn from the market in March 2018 [82].

Frexalimab is a novel anti-CD40L (CD154) mAb currently under investigation in MS. By binding to CD40L, a key molecule expressed on activated T cells, frexalimab disrupts the CD40-CD40L interaction essential for T cell and B cell activation, proliferation, and differentiation. This blockade inhibits germinal center formation, reduces the production of pathogenic antibodies and proinflammatory cytokines, and modulates the activation of innate immune cells, thereby suppressing the adaptive immune responses driving MS pathogenesis. Clinical trials have demonstrated promising results, showing a significant reduction in new gadolinium-enhancing T1 lesions and new or enlarging T2 lesions on MRI. With its unique mechanism of action targeting T cell–B cell interactions, frexalimab offers a potential alternative or complementary strategy to existing MS therapies [83,84].

Foralumab, a fully human anti-CD3 antibody, was very recently tested in an open-label study, which enrolled 10 patients with non-active secondary progressive MS (naSPMS) who continued to progress on B-cell therapy. Foralumab was administered intranasally for six months. The treatment showed efficacy as no EDSS score progression was registered, and three of four patients treated continuously for 12 months experienced an improvement in EDSS. Six of ten patients had improvement in fatigue on the Modified Fatigue Impact Scale (MFIS) scale. No treatment-related serious adverse events (SAEs) or severe AEs were reported, and no new T2 lesions were observed on MRI. Changes in peripheral blood gene expression occurred as early as three months and affected antigen presentation, interferon responses, and regulatory pathways in multiple cell types, including FoxP3^+^ Tregs, CD4^+^ Tcm cells, CD8^+^ Tem cells, CD14^+^ and CD16^+^ monocytes, and B cells. TGFβ expression was increased across multiple cell subsets [85].

Tolebrutinib, a BTK inhibitor (BTKi), has been tested in three phase III clinical trials, including RRMS (NCT04410991, GEMINI2), primary progressive MS (PPMS) (NCT04458051, PERSEUS), and non-relapsing SPMS (NRSPMS) (NCT04411641, HERCULES) patients. According to very recently published data, non-relapsing secondary progressive MS patients treated with tolebrutinib displayed significantly lower disability progression compared to their placebo counterparts [64]. However, tolebrutinib failed to prove its superiority compared to teriflunomide in reducing ARR among patients with RRMS [65]. Minor bleeding [65] and significantly (3 times higher than normal) higher alanine transferase levels [64] were the most common adverse events. Moreover, very recently, the results of a double-blind, randomized, placebo-controlled phase II trial (FENopta, NCT05119569) conducted in relapsing MS patients were reported. According to the authors, fenebrutinib showed efficacy in reducing the number of new T1 Gd+ lesions compared to placebo. The major safety concerns were increased hepatic enzyme elevations, headache, and nasopharyngitis, while no serious adverse events or deaths occurred [66].

### 2.2. Neuromyelitis Optica Spectrum Disorder

Neuromyelitis optica spectrum disorder (NMOSD) is an autoimmune astrocytopathic disease of the CNS, primarily mediated by aquaporin-4 (AQP4) immunoglobulin G (IgG) autoantibodies, leading to astrocyte injury, demyelination, and severe neurological deficits [86]. Unlike MS, where autoreactive T cells and diverse immune mechanisms drive the pathophysiological processes, NMOSD is characterized by a more targeted autoimmune attack on AQP4, the principal water channel protein highly expressed on astrocytes, particularly in the optic nerves, spinal cord, and periventricular regions. The pathogenic cascade begins when circulating AQP4-IgG autoantibodies bind to AQP4 receptors on astrocytes, triggering several downstream effector mechanisms—CDC, ADCC, and astrocyte internalization of AQP4—leading to astrocyte dysfunction and death. Complement activation results in the formation of the membrane attack complex (MAC), which directly lyses astrocytes and releases proinflammatory mediators that amplify tissue damage. ADCC is mediated by natural killer (NK) cells and other immune cells that bind to the Fc region of AQP4-IgG, resulting in astrocyte destruction. Furthermore, the loss of AQP4-expressing astrocytes disrupts water homeostasis, glutamate regulation, and potassium buffering within the CNS, contributing to neuronal dysfunction and demyelination. Interleukin (IL)-6 is a multifunctional, soluble cytokine considered to play a central role in NMOSD pathophysiology by promoting the differentiation, survival, and antibody production of plasmablasts, which are key drivers of AQP4-IgG autoantibody production. The preferential localization of AQP4 in specific CNS regions explains the characteristic clinical manifestations of NMOSD, including optic neuritis, transverse myelitis, and area postrema syndrome. Pathogenic anti-AQP4 autoantibodies are found in over two-thirds of patients with NMOSD, while they are not detected in patients with MS. Recent advances in understanding its pathogenic mechanisms have led to the development of targeted mAb therapies against NMOSD, namely complement inhibition (eculizumab, ravulizumab), IL-6 receptor blockade (satralizumab), and B cell depletion (rituximab), thereby selectively modulating the autoantibody-mediated astrocyte injury and preventing relapses and disability progression in patients with NMOSD [87,88,89]. In NMOSD, mAbs target key pathways, as detailed in Figure 1 and Table 2.

#### 2.2.1. mAbs Targeting IL-6

Satralizumab is a humanized IgG2 mAb that binds to the IL-6 receptor (IL-6R), thereby inhibiting the downstream activation of IL-6-mediated signaling pathways involved in the differentiation, survival, and AQP4-IgG antibody production of plasmablasts. Furthermore, satralizumab’s unique pH-dependent binding allows it to recycle through the endosomal pathway, prolonging its half-life and enabling sustained IL-6 receptor blockade. It has been approved for seropositive AQP4-IgG NMOSD, while elevated liver enzymes, infections, and neutropenia represent the most common side effects [90].

Tocilizumab is another humanized IgG1 mAb targeting IL-6R and blocking the IL-6 pathway. Several case series and reports suggest that tocilizumab treatment could be a valuable treatment option for NMOSD [91,92]. A recent meta-analysis reported that tocilizumab is more effective in AQP4-IgG-positive NMOSD patients; the reduction in ARR ratio was associated with gender, race, and dosage, while the effectiveness of reducing EDSS score was not related to these factors. Tocilizumab treatment is associated with mild adverse events and very rare severe adverse reactions (facial cellulitis) [93]. Despite the promising evidence supporting its clinical effectiveness and safety in NMOSD therapy, tocilizumab remains an off-label treatment.

#### 2.2.2. Complement Inhibition

Eculizumab, a humanized monoclonal IgG2/4κ antibody, blocks the formation of the MAC and subsequent astrocyte lysis via the inhibition of the terminal complement pathway. It is the first FDA-approved mAb for complement-mediated diseases, including paroxysmal nocturnal hemoglobinuria (2007) and atypical hemolytic uremic syndrome (2011). In 2018, it was additionally approved for generalized myasthenia gravis (gMG) with positive anti-acetylcholine receptor antibodies (anti-AchR Abs), as it aids in the preservation of the postsynaptic membrane [53]. Clinical evidence from the PREVENT trial established eculizumab as an effective therapy for reducing relapse rates in AQP4-IgG seropositive NMOSD patients. The therapeutic scheme, the same as that for gMG, involves injections of 1200 mg twice monthly, after the initial treatment period of 5 weeks [53]. Side effects include infections, arthralgia, back pain, dizziness, and diarrhea, while the most significant safety concern is an increased risk of meningococcal infections, necessitating prior vaccination with the Neisseria meningitidis vaccine and regular monitoring [53]. Eculizumab received approval for NMO therapy in 2019 [94].

Ravulizumab, a humanized monoclonal IgG2/4 antibody, is a modified derivative of eculizumab, genetically engineered to enhance its duration of action and reduce the frequency of infusions. By incorporating specific amino acid substitutions in the Fc region, ravulizumab exhibits increased affinity for the neonatal Fc receptor (FcRn), resulting in prolonged recycling and reduced clearance from circulation. Ravulizumab has recently been approved for NMOSD treatment [95,96]. Compared to eculizumab, ravulizumab shows a more prolonged duration of action [101]. Ravulizumab is indicated as an add-on to standard therapy for the treatment of adult patients with generalized myasthenia gravis (gMG) who are anti-acetylcholine receptor (AChR) antibody-positive (see below), as well as for the treatment of adult patients with NMOSD who are anti-aquaporin-4 (AQP4) antibody-positive. Mechanistically, ravulizumab inhibits the terminal complement cascade by binding to C5, preventing its cleavage into C5a and C5b, thereby blocking the formation of the MAC and subsequent inflammation and tissue damage. Notably, although head-to-head trials are lacking, in a Bayesian network meta-analysis, ravulizumab monotherapy was associated with a reduced relapse rate compared to inebilizumab or sartralizumab monotherapies [102], suggesting its potential superiority.

#### 2.2.3. Other B Cell Depletion Therapies

Inebilizumab is a humanized IgG1 mAb targeting CD19, a surface antigen expressed on a broad range of B cells, including plasmablasts and some plasma cells. Through binding to CD19, inebilizumab induces B cell depletion through mechanisms such as ADCC and ADCP. The decrease in the B cell subpopulation is noticed from the first 4 weeks after initiation of treatment, while the exact time of action and clinical effectiveness is unknown [103]. It is administered via intravenous infusion. Inebilizumab’s efficacy and safety have been documented by important clinical trials [97,98], leading to FDA approval for patients with NMOSD and positive anti-AQP4 antibodies [99], and also very recently (April 2025) for IgG4-related disease (IgG4-RD) [100,104].

## 3. Neurodegenerative Diseases of the CNS

### 3.1. Alzheimer’s Disease

**Figure 2:**
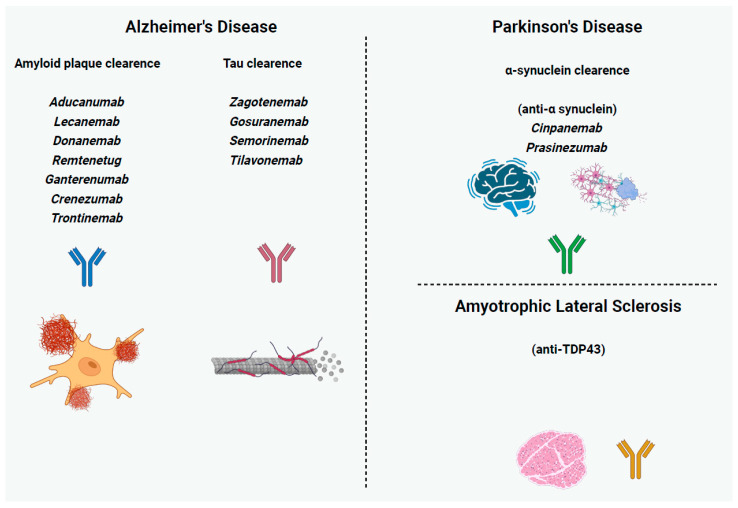
**Monoclonal antibodies targeting pathological protein aggregates in neurodegenerative diseases.** In Alzheimer’s disease (AD), therapeutic antibodies focus on amyloid plaque clearance (aducanumab, lecanemab, donanemab, remternetug, gantenerumab, crenezumab, trontinemab) and tau clearance (zagotenemab, gosuranemab, semorinemab, tilavonemab). In Parkinson’s disease (PD), investigational antibodies (cinpanemab, prasinezumab) are directed against α-synuclein clearance. In amyotrophic lateral sclerosis (ALS), therapeutic strategies under development aim at antibodies against TDP-43 aggregates.

Alzheimer’s disease (AD) is the most prevalent neurodegenerative disorder characterized by progressive cognitive decline, ultimately leading to profound dementia [105]. The core neuropathological hallmarks of AD include extracellular amyloid plaques composed of aggregated amyloid-beta (Aβ) peptides and intracellular neurofibrillary tangles (NFTs) formed by hyperphosphorylated tau protein, along with synaptic dysfunction and neuronal loss. According to the amyloid cascade hypothesis, the accumulation of Aβ peptides, derived from the proteolytic processing of amyloid precursor protein (APP) by β-secretase 1 (BACE1) and γ-secretase, initiates a cascade of events leading to tau hyperphosphorylation, tangle formation, neuroinflammation, and neuronal death. Specifically, Aβ oligomers, rather than insoluble plaques, are considered the most neurotoxic species, disrupting synaptic plasticity, impairing neuronal function, and triggering microglial activation and proinflammatory cytokine release. Hyperphosphorylated tau accumulates within neurons, disrupting microtubule function, impairing axonal transport, and forming NFTs, which correlate closely with cognitive decline. Neuroinflammation, involving activated microglia and astrocytes, contributes to neuronal damage through the release of inflammatory mediators and reactive oxygen species (ROS). Genetic risk factors, such as mutations in APP, presenilin 1 (PSEN1), and presenilin 2 (PSEN2), increase Aβ production, while the ε4 allele of apolipoprotein E (APOE) mainly impairs Aβ clearance. These mechanisms have prompted the development of mAb therapies targeting Aβ plaques and tau, which until recently have largely failed, possibly due to interventions occurring late in the disease when irreversible synapse or neuron loss has already occurred [106,107,108,109]. Next, we describe mAbs according to AD-related pathophysiological targets (Figure 2 and Table 3).

#### 3.1.1. Anti-Amyloid mAbs

Aducanumab, a human IgG1 mAb, selectively binds to the N-terminal epitope of the Aβ42 peptide, preferentially targeting fibrillar Aβ aggregates [110]. Granted accelerated FDA approval in June 2021 [111,112], it became the first Aβ-directed mAb and disease-modifying therapy for AD. Aducanumab is indicated for early AD patients with MCI or mild dementia with confirmed presence of Aβ in the brain, either via PET or CSF analysis. Based on the related clinical trials, it is delivered intravenously every four weeks with a target dose of 10 mg/kg [110].

Lecanemab is a humanized IgG1 mAb derived from the murine mAb158, designed to selectively target Aβ protofibrils [113]. The agent received accelerated approval by the FDA in January 2023, with full approval granted in July 2023, for MCI or mild dementia with confirmed AD pathology [114]. It is administered intravenously at a fixed dose of 10 mg/kg every two weeks without the need for weight-based titration. Beyond the United States, the drug has also recently secured approval in Japan and the European Union [113].

Donanemab is another humanized IgG1 mAb derived from the murine mE8-IgG2a antibody, specifically designed to recognize the N-terminal pyroglutamate form of Aβ, thereby binding to deposited Aβ and promoting the clearance of Aβ plaques by microglia [127]. Donanemab was fully approved by the FDA in July 2024 for confirmed early AD [115]. It is delivered intravenously once a month, at a fixed dose of 350 mg/20 mL. On 24 July 2025, the Committee for Medicinal Products for Human Use (CHMP), after strict re-examination, adopted a positive opinion, recommending the granting of a marketing authorization for the donamemab, intended for the treatment of early symptomatic Alzheimer’s disease, in adults who are apolipoprotein E ε4 (ApoE ε4) non-carriers or heterozygotes.

Remternetug is a mAb targeting N3pG-AB, with a high affinity for deposited Aβ plaques, sharing a similar mechanism of action with donanemab but with improved pharmacokinetic properties, allowing for potential subcutaneous administration and less frequent dosing [116]. Currently, remternetug is being evaluated in several phase III clinical trials for patients with early symptomatic AD.

Gantenerumab is the first human IgG1 anti-Aβ mAb that has advanced into clinical development. It binds to both the N-terminal regions and central amino acid sequences of Aβ, thereby facilitating the microglia-mediated Aβ clearance of aggregated Aβ forms [117,118].

Crenezumab is a humanized IgG4 anti-Aβ mAb targeting Aβ oligomers. In two phase III clinical trials, crenezumab was well-tolerated but failed to affect clinical decline in patients with prodromal to mild AD [119].

Trontinemab is another anti-Aβ mAb under investigation. It is based on gantenerumab but is fused with a brain shuttle module that binds to transferrin receptors, facilitating its active transport across the BBB into the brain [120,121]. By improving CNS penetration, trontinemab aims to achieve greater amyloid plaque clearance at lower systemic doses compared to conventional mAbs. This innovative therapeutic strategy aims to optimize efficacy while reducing the risk of peripheral side effects [120]. Currently, trontinemab is being investigated in phase II trials to assess its safety, tolerability, and preliminary efficacy in individuals with early symptomatic AD.

The anti-amyloid mAbs differ in their exact mechanism of action, the Aβ form they selectively target, the MRI monitoring protocols for amyloid-related imaging abnormalities (ARIA), titration schedules, and interrupted versus continuing treatment [9]. Importantly, the clinical efficacy of anti-amyloid mAbs in AD requires careful interpretation of statistical versus clinically meaningful outcomes. For instance, while lecanemab is associated with a “27% greater slowing” in cognitive decline compared to placebo as measured on the CDR-SB scale, this change represents only 2.5% of the total scale range (0.45 points on an 18-point scale), constituting approximately one-third of the minimal clinically significant difference, as reported by Andrews and colleagues.

A study that evaluated the cost-effectiveness of lecanemab showed that standard care alone was the most cost-effective approach. Importantly, it was estimated that this treatment, following CSF testing, could become cost-effective if the annual price of lecanemab fell below USD 5100. These findings were consistent across variations in diagnostic accuracy, treatment discontinuation, and adverse event rates [128].

#### 3.1.2. Anti-Tau mAbs

Zagotenemab is a humanized mAb that selectively binds the extracellular, misfolded, aggregated tau protein [129]. No clinical benefit was observed in patients with early symptomatic AD in the phase II PERISCOPE-ALZ trial, in terms of slowing functional or cognitive decline compared to placebo. Furthermore, no significant benefits were demonstrated in neurofilament light chain (Nfl) levels, volumetric brain MRI, and flortaucipir PET imaging, indicating a lack of measurable disease-modifying effects [122].

Gosuranemab, a humanized IgG4 mAb, targets abnormal forms of tau protein or soluble oligomers. A phase II trial indicated no significant clinical efficacy in cognitive and functional measures, compared to placebo in early AD [130], but also in progressive supranuclear palsy (PSP), another neurodegenerative disease characterized by abnormal tau accumulation [123].

Semorinemab is another humanized IgG4 mAb targeting tau protein that has been studied in prodromal to mild AD phase II clinical trials. In this study, semorinemab was well-tolerated but failed to slow the clinical progression of AD [124]. In another phase II study in patients with mild to moderate AD, although semorinemab demonstrated benefit in cognitive performance as measured by the Alzheimer’s Disease Assessment Scale-Cognitive Subscale (ADAS-Cog11) compared to placebo, it did not affect global cognition or functional outcomes [125].

Tilavonemab, a humanized IgG4 mAb, acts against the abnormal extracellular forms of tau protein. In a phase II trial, tilavonemab had a good safety profile but did not show significant clinical benefits compared to placebo [126].

### 3.2. Parkinson’s Disease

Parkinson’s disease (PD) is the second most prevalent neurodegenerative disorder, primarily characterized by the loss of dopaminergic neurons in the substantia nigra pars compacta (SNpc) and the accumulation of misfolded α-synuclein protein in intraneuronal inclusions known as Lewy bodies [131]. Protein misfolding and aggregation, mitochondrial dysfunction, oxidative stress, neuroinflammation, and impaired cellular clearance systems contribute to its pathophysiology. The hallmark α-synuclein pathology begins with the misfolding of this normally soluble protein into β-sheet-rich conformations that aggregate into oligomers, protofibrils, and eventually mature fibrils, constituting Lewy bodies. These α-synuclein aggregates exhibit prion-like properties, spreading from cell to cell and propagating pathology throughout the brain in a stereotypical pattern described by Braak staging. Mitochondrial dysfunction, characterized by reduced complex I activity, increased ROS production, and impaired mitophagy, contributes significantly to neuronal vulnerability. Neuroinflammation, mediated by activated microglia and astrocytes, releases proinflammatory cytokines and ROS that further exacerbate neurodegeneration. Genetic factors, including mutations in SNCA (α-synuclein), LRRK2, GBA, and PRKN genes, influence disease susceptibility and progression through various pathways affecting protein homeostasis, mitochondrial function, and lysosomal autophagy systems [132,133,134].

Our growing understanding of PD pathophysiology contributes to the exploration of the role of mAbs targeting α-synuclein aggregates (cinpanemab, prasinezumab) that could inhibit protein misfolding, block cell-to-cell transmission, and modify disease progression [8]. However, until now, clinical trials have yielded modest results, highlighting the challenges related to the development of disease-modifying therapies for this complex neurodegenerative disease. Next, we describe mAbs according to PD-related pathophysiological mechanism targets (Figure 2 and Table 4).

#### Anti-a-Synuclein mAbs

Cinpanemab is a humanized IgG1 mAb targeting aggregated forms of α-synuclein. Although cinpanemab could effectively bind pathological α-synuclein, no significant clinical benefits were observed in patients with early PD compared to placebo [135]. In addition, data derived from the phase II SPARK trial, biomarker analyses, including imaging and fluid markers, did not reveal significant results, highlighting the ongoing challenges in developing effective immunotherapies for synucleinopathies [136].

Prasinezumab is a humanized monoclonal antibody that selectively binds aggregated α-synuclein at the C-terminal of the protein [137]. Similarly to cinpanemab, published data from the phase II trial PASADENA show that prasinezumab therapy had no significant effect on global or imaging measures of PD progression as compared with placebo and was associated with infusion reactions [138].

## 4. Neurodegenerative Diseases of the CNS and PNS

### 4.1. Amyotrophic Lateral Sclerosis

Amyotrophic lateral sclerosis (ALS) is a neurodegenerative disorder of the CNS and peripheral nervous system (PNS) characterized by the selective loss of motor neurons in the brain and spinal cord, leading to muscle weakness and ultimately respiratory failure [140]. The incidence of ALS is estimated at between 0.6 and 3.8 per 100,000 person-years [141,142], while in Europe, the incidence of ALS is higher, ranging from 2.1 to 3.8 per 100,000 person-years [141,143]. The pathophysiology of ALS is multifactorial, involving genetic, environmental, and cellular mechanisms that converge to cause motor neuron dysfunction and death. Key mechanisms include protein misfolding and aggregation, glutamate excitotoxicity, oxidative stress, mitochondrial dysfunction, neuroinflammation, impaired axonal transport, and RNA processing defects. Mutations in genes such as superoxide dismutase 1 (SOD1), TAR DNA-binding protein 43 (TDP-43), fusion protein (FUS), chromosome 9 open reading frame 72 (C9orf72), and others account for approximately 10% of ALS cases (familial ALS), while the remaining 90% are classified as sporadic. Protein misfolding and aggregation, particularly of TDP-43 and SOD1, are prominent features in both familial and sporadic ALS. Glutamate excitotoxicity, resulting from impaired glutamate transport and excessive stimulation of glutamate receptors, as well as oxidative stress, resulted from mitochondrial dysfunction and increased ROS production, damaged cellular macromolecules, and exacerbated neurodegeneration. Neuroinflammation, mediated by activated microglia and astrocytes and the accompanying release of proinflammatory cytokines, mainly contributes to motor neuron injury [144,145,146]. While tofersen, an antisense oligonucleotide, is approved for SOD1-mutated ALS, mAb approaches are currently being explored to regulate neuroinflammation and protein aggregation, aiming to slow disease progression by targeting specific pathways [147].

#### Anti-TAR DNA-Binding Protein 43 (TDP43) mAbs

Phosphorylated TDP-43 at serines 409/410 (pS409/410) represents a pathological hallmark of frontotemporal dementia (FTD) and ALS [148]. Recently, novel rabbit mAbs with high specificity and sensitivity for pS409/410-TDP-43 were developed, which could effectively bind to pathological TDP-43 inclusions in the brain tissue of human patients with FTD/ALS [139] (Figure 2 and Table 4). These findings establish these mAbs as valuable tools for advancing research on TDP-43 pathology and pave the way for future therapeutic investigations.

## 5. Conclusions

Monoclonal antibodies have emerged as innovative agents in the treatment of CNS diseases by enabling selective immunopathological and molecular targeting. Their application in MS and NMOSD has established them as cornerstones of therapy, while in neurodegenerative diseases like Alzheimer’s and Parkinson’s, they offer a glimpse into future disease-modifying strategies. Despite substantial progress, challenges remain, including treatment costs, safety concerns, and limited CNS bioavailability. Ongoing innovation in antibody bioengineering, along with the integration of predictive biomarkers, holds promise for enhancing therapeutic specificity and patient outcomes. Future research must address optimal patient selection, long-term safety, and synergistic treatment approaches to fully realize the potential of mAb-based therapies in neurology, while Multi-Omics strategies already give a new, expanding, and ongoing utility of established mAb-based therapies towards precision medicine.

## Figures and Tables

**Table 1:** Monoclonal antibodies in multiple sclerosis (MS).

Mechanism of Action	Medication	Disease	Clinical Trial Registration Number	Type of Study	Status	References
B cell depletion (anti-CD20)	Rituximab	MS (RRMS, SPMS, PPMS)	NCT00087529, NCT02932304	Phase II/III, randomized, double-blind	Completed	[38,39,40,41]
	Ocrelizumab	RRMS, PPMS	NCT01247324, NCT01194570	Phase III, randomized, double-blind	Approved (FDA/EMA)	[41,42,43]
	Ofatumumab	RRMS	NCT02077361	Phase III, randomized, double-blind	Approved (FDA/EMA)	[44,45]
	Ublituximab	RRMS	NCT03277261, NCT03277248	Phase III, randomized, double-blind	Approved (FDA)	[44,46]
Inhibition of leukocyte migration (anti-α4 integrin)	Natalizumab	RRMS, POMS	NCT00027300	Phase III, randomized, double-blind	Approved (FDA/EMA)	[42,47,48,49,50,51,52,53]
Immune reset (anti-CD52)	Alemtuzumab	RRMS	NCT00548405, NCT00530348	Phase III, randomized, open-label	Approved (restricted use EMA)	[52,54,55,56,57,58]
Remyelination (LINGO-1 inhibition)	Opicinumab	MS (RRMS, acute optic neuritis)	NCT01721161, NCT01894684	Phase II, randomized, double-blind	Completed-Discontinued	[59,60]
T-B cell interaction blockade (anti-CD40L)	Frexalimab	MS	NCT04879628	Phase II, randomized, double-blind	Active, not recruiting	[61,62]
Anti-CD3 mAb	Foralumab	SPMS	NCT04594085	Open-label	Completed	[63]
BTKi	Tolebrutinib	RRMS	NCT04410991	Phase III, randomized, double-blind	Completed	[64,65]
		PPMS	NCT04458051			
		SPMS	NCT04411641			
	Fenebrutinib	RRMS	NCT05119569	Phase II, randomized, double-blind	Active, not recruiting	[66]

MS: multiple sclerosis; RRMS: relapsing–remitting multiple sclerosis; SPMS: secondary progressive multiple sclerosis; PPMS: primary progressive multiple sclerosis; POMS: pediatric-onset multiple sclerosis; FDA: Food and Drug Administration; EMA: European Medicines Agency.

**Table 2:** Monoclonal antibodies in neuromyelitis optica spectrum disorder (NMOSD).

Mechanism of Action	Medication	Disease	Clinical Trial Registration Number	Type of Study	Status	References
IL-6 receptor blockade	Satralizumab	NMOSD (AQP4-IgG+)	NCT02073279, NCT02028884	Phase III, randomized, double-blind	Approved (FDA/EMA)	[90]
	Tocilizumab	NMOSD (AQP4-IgG+)	NCT02028884	Phase II, open-label, extension	Off-label	[91,92,93]
Complement inhibition (C5 blockade)	Eculizumab	NMOSD (AQP4-IgG+)	NCT01892345	Phase III, randomized, double-blind	Approved (FDA/EMA)	[94]
	Ravulizumab	NMOSD (AQP4-IgG+)	NCT03330418	Phase III, randomized, double-blind	Approved (FDA/EMA)	[95,96]
B cell depletion (anti-CD19)	Inebilizumab	NMOSD (AQP4-IgG+)	NCT02200770 (NMOmentum)	Phase II/III, randomized, double-blind	Approved (FDA)	[97,98,99,100]

NMOSD: neuromyelitis optica spectrum disorder; AQP4: aquaporin 4; FDA: Food and Drug Administration; EMA: European Medicines Agency.

**Table 3:** Monoclonal antibodies in Alzheimer’s disease (AD).

Mechanism of Action	Medication	Disease	Clinical Trial Registration Number	Type of Study	Status	References
Anti-amyloid (Aβ fibrils/plaques)	Aducanumab	AD (MCI, mild dementia)	NCT02477800NCT02484547	Phase III, randomized, double-blind	FDA accelerated approval (2021)	[110,111,112]
Anti-amyloid (Aβ protofibrils)	Lecanemab	AD (early AD)	NCT01767311NCT03887455	Phase II/III, randomized, double-blind	FDA full approval (2023), EMA/Japan approved	[113,114]
Anti-amyloid (Aβ pyroglutamate)	Donanemab	AD (early AD, ApoE ε4 non-carriers/heterozygotes)	NCT04437511	Phase III, randomized, double-blind	FDA full approval (2024), EMA positive opinion (2025)	[115]
Anti-amyloid (N3pG-Aβ)	Remternetug	AD (early AD)	NCT05869306NCT05869319	Phase III, randomized, double-blind	Ongoing	[116]
Anti-amyloid (Aβ plaques)	Gantenerumab	AD (early AD)	NCT03443973NCT03444870	Phase III, randomized, double-blind	Terminated	[117,118]
Anti-amyloid (Aβ oligomers)	Crenezumab	AD (prodromal to mild)	NCT02670083NCT03114657	Phase III, randomized, double-blind	Terminated	[119]
Anti-amyloid with brain shuttle	Trontinemab	AD (early AD)	NCT04639050	Phase II, randomized, double-blind	Active, not recruiting	[120,121]
Anti-tau	Zagotenemab	AD (early symptomatic)	NCT03019536	Phase II, randomized, double-blind	Completed	[122]
Anti-tau	Gosuranemab	AD	NCT03352557NCT03068468	Phase II, randomized, double-blind	Terminated	[123]
Anti-tau	Semorinemab	AD (mild to moderate)	NCT03828747NCT03352557	Phase II, randomized, double-blind	Completed	[124,125]
Anti-tau	Tilavonemab	AD	NCT02880956	Phase II, randomized, double-blind	Completed	[126]

AD: Alzheimer’s disease; MCI: mild cognitive impairment; FDA: Food and Drug Administration; EMA: European Medicines Agency.

**Table 4:** Monoclonal antibodies in Parkinson’s disease (PD) and amyotrophic lateral sclerosis (ALS).

Mechanism of Action	Medication	Disease	Clinical Trial Registration Number	Type of Study	Status	References
Anti-α-synuclein	Cinpanemab	PD (early PD)	NCT03318523 (SPARK)	Phase II, randomized, double-blind	Terminated	[135,136]
Anti-α-synuclein	Prasinezumab	PD (early PD)	NCT03100149 (PASADENA)	Phase II, randomized, double-blind	Active, not recruiting	[137,138]
Anti-TDP-43 (pS409/410)	Experimental mAbs	ALS/FTD	-	Preclinical/exploratory	Ongoing (preclinical)	[139]

## References

[1] Smith S.L. (1996). Ten Years of Orthoclone OKT3 (Muromonab-CD3): A Review. J. Transpl. Coord..

[2] Salles G., Barrett M., Foà R., Maurer J., O’Brien S., Valente N., Wenger M., Maloney D.G. (2017). Rituximab in B-Cell Hematologic Malignancies: A Review of 20 Years of Clinical Experience. Adv. Ther..

[3] Skarlis C., Kotsari M., Anagnostouli M. (2025). Advancing Treatment in Pediatric Multiple Sclerosis: The Promise of B-Cell-Targeting Therapies. Int. J. Mol. Sci..

[4] Skarlis C., Argyriou E., Mavragani C.P. (2020). Lymphoma in Sjögren’s Syndrome: Predictors and Therapeutic Options. Curr. Treat. Options Rheum..

[5] Skarlis C., Marketos N., Mavragani C.P. (2019). Biologics in Sjögren’s Syndrome. Pharmacol. Res..

[6] Papadopoulos V.E., Skarlis C., Evangelopoulos M.-E., Mavragani C.P. (2021). Type I Interferon Detection in Autoimmune Diseases: Challenges and Clinical Applications. Expert. Rev. Clin. Immunol..

[7] Elices M.J. (2003). Natalizumab. Elan/Biogen. Curr. Opin. Investig. Drugs.

[8] Kharel S., Ojha R. (2023). Future of Monoclonal Antibody Therapy in Parkinson’s Disease. Ann. Neurosci..

[9] Cummings J., Osse A.M.L., Cammann D., Powell J., Chen J. (2024). Anti-Amyloid Monoclonal Antibodies for the Treatment of Alzheimer’s Disease. BioDrugs.

[10] Lublin F.D., Reingold S.C., Cohen J.A., Cutter G.R., Sørensen P.S., Thompson A.J., Wolinsky J.S., Balcer L.J., Banwell B., Barkhof F. (2014). Defining the Clinical Course of Multiple Sclerosis. Neurology.

[11] Reich D.S., Lucchinetti C.F., Calabresi P.A. (2018). Multiple Sclerosis. N. Engl. J. Med..

[12] Waubant E., Lucas R., Mowry E., Graves J., Olsson T., Alfredsson L., Langer-Gould A. (2019). Environmental and Genetic Risk Factors for MS: An Integrated Review. Ann. Clin. Transl. Neurol..

[13] Skarlis C., Anagnostouli M. (2020). The Role of Melatonin in Multiple Sclerosis. Neurol. Sci..

[14] Manna I., De Benedittis S., Porro D. (2024). A Comprehensive Examination of the Role of Epigenetic Factors in Multiple Sclerosis. Int. J. Mol. Sci..

[15] Munger K.L., Chitnis T., Ascherio A. (2009). Body Size and Risk of MS in Two Cohorts of US Women. Neurology.

[16] Mikaeloff Y., Caridade G., Tardieu M., Suissa S., KIDSEP Study Group (2007). Parental Smoking at Home and the Risk of Childhood-Onset Multiple Sclerosis in Children. Brain.

[17] Banwell B., Krupp L., Kennedy J., Tellier R., Tenembaum S., Ness J., Belman A., Boiko A., Bykova O., Waubant E. (2007). Clinical Features and Viral Serologies in Children with Multiple Sclerosis: A Multinational Observational Study. Lancet Neurol..

[18] Hedström A.K., Sundqvist E., Bäärnhielm M., Nordin N., Hillert J., Kockum I., Olsson T., Alfredsson L. (2011). Smoking and Two Human Leukocyte Antigen Genes Interact to Increase the Risk for Multiple Sclerosis. Brain.

[19] Bjornevik K., Münz C., Cohen J.I., Ascherio A. (2023). Epstein–Barr Virus as a Leading Cause of Multiple Sclerosis: Mechanisms and Implications. Nat. Rev. Neurol..

[20] Anagnostouli M., Anagnostoulis G., Katsavos S., Panagiotou M., Kararizou E., Davaki P. (2014). HLA-DRB1 15:01 and Epstein-Barr Virus in a Multiple Sclerosis Patient with Psoriasis, Nasopharyngeal and Breast Cancers. Lessons for Possible Hidden Links for Autoimmunity and Cancer. J. Neurol. Sci..

[21] Sawcer S., Hellenthal G., Pirinen M., Spencer C.C.A., Patsopoulos N.A., Moutsianas L., Dilthey A., Su Z., International Multiple Sclerosis Genetics Consortium, Wellcome Trust Case Control Consortium 2 (2011). Genetic Risk and a Primary Role for Cell-Mediated Immune Mechanisms in Multiple Sclerosis. Nature.

[22] International Multiple Sclerosis Genetics Consortium (2019). Multiple Sclerosis Genomic Map Implicates Peripheral Immune Cells and Microglia in Susceptibility. Science.

[23] Anagnostouli M., Artemiadis A., Gontika M., Skarlis C., Markoglou N., Katsavos S., Kilindireas K., Doxiadis I., Stefanis L. (2020). HLA-DPB1*03 as Risk Allele and HLA-DPB1*04 as Protective Allele for Both Early- and Adult-Onset Multiple Sclerosis in a Hellenic Cohort. Brain Sci..

[24] Patsopoulos N.A., Barcellos L.F., Hintzen R.Q., Schaefer C., van Duijn C.M., Noble J.A., Raj T., Gourraud P.-A., IMSGC, ANZgene (2013). Fine-Mapping the Genetic Association of the Major Histocompatibility Complex in Multiple Sclerosis: HLA and Non-HLA Effects. PLoS Genet..

[25] Patsopoulos N.A. (2018). Genetics of Multiple Sclerosis: An Overview and New Directions. Cold Spring Harb. Perspect. Med..

[26] Skarlis C., Markoglou N., Gontika M., Artemiadis A., Pons M.-R., Stefanis L., Dalakas M., Chrousos G., Anagnostouli M. (2024). The Impact of HLA-DRB1 Alleles in a Hellenic, Pediatric-Onset Multiple Sclerosis Cohort: Implications on Clinical and Neuroimaging Profile. Neurol. Sci..

[27] Moutsianas L., Jostins L., Beecham A.H., Dilthey A.T., Xifara D.K., Ban M., Shah T.S., Patsopoulos N.A., Alfredsson L., Anderson C.A. (2015). Class II HLA Interactions Modulate Genetic Risk for Multiple Sclerosis. Nat. Genet..

[28] Barcellos L.F., Sawcer S., Ramsay P.P., Baranzini S.E., Thomson G., Briggs F., Cree B.C.A., Begovich A.B., Villoslada P., Montalban X. (2006). Heterogeneity at the HLA-DRB1 Locus and Risk for Multiple Sclerosis. Hum. Mol. Genet..

[29] Sawcer S., Franklin R.J.M., Ban M. (2014). Multiple Sclerosis Genetics. Lancet Neurol..

[30] Skarlis C., Papadopoulos V., Raftopoulou S., Mavragani C.P., Evangelopoulos M.-E. (2023). B-Cell Activating Factor Gene Variants in Multiple Sclerosis: Possible Associations with Disease Susceptibility among Females. Clin. Immunol..

[31] Ntellas P., Dardiotis E., Sevdali E., Siokas V., Aloizou A.-M., Tsinti G., Germenis A.E., Hadjigeorgiou G.M., Eibel H., Speletas M. (2020). TNFRSF13C/BAFFR P21R and H159Y Polymorphisms in Multiple Sclerosis. Mult. Scler. Relat. Disord..

[32] Steri M., Orrù V., Idda M.L., Pitzalis M., Pala M., Zara I., Sidore C., Faà V., Floris M., Deiana M. (2017). Overexpression of the Cytokine BAFF and Autoimmunity Risk. N. Engl. J. Med..

[33] Arneth B.M. (2019). Impact of B Cells to the Pathophysiology of Multiple Sclerosis. J. Neuroinflammation.

[34] Compston A., Coles A. (2008). Multiple Sclerosis. Lancet.

[35] Artemiadis A.K., Anagnostouli M.C. (2010). Apoptosis of Oligodendrocytes and Post-Translational Modifications of Myelin Basic Protein in Multiple Sclerosis: Possible Role for the Early Stages of Multiple Sclerosis. Eur. Neurol..

[36] Stampanoni Bassi M., Iezzi E., Centonze D. (2022). Multiple Sclerosis: Inflammation, Autoimmunity and Plasticity. Handb. Clin. Neurol..

[37] Mazzeo A.C., Calabresi L., Damato V., Spagni G., Massacesi L., Mariottini A. (2025). CD20+ T Cells in Multiple Sclerosis: From Pathogenesis to Treatment-Induced Depletion. Int. J. Mol. Sci..

[38] Margoni M., Preziosa P., Filippi M., Rocca M.A. (2022). Anti-CD20 Therapies for Multiple Sclerosis: Current Status and Future Perspectives. J. Neurol..

[39] Salzer J., Svenningsson R., Alping P., Novakova L., Björck A., Fink K., Islam-Jakobsson P., Malmeström C., Axelsson M., Vågberg M. (2016). Rituximab in Multiple Sclerosis: A Retrospective Observational Study on Safety and Efficacy. Neurology.

[40] Alping P., Frisell T., Novakova L., Islam-Jakobsson P., Salzer J., Björck A., Axelsson M., Malmeström C., Fink K., Lycke J. (2016). Rituximab versus Fingolimod after Natalizumab in Multiple Sclerosis Patients. Ann. Neurol..

[41] Granqvist M., Boremalm M., Poorghobad A., Svenningsson A., Salzer J., Frisell T., Piehl F. (2018). Comparative Effectiveness of Rituximab and Other Initial Treatment Choices for Multiple Sclerosis. JAMA Neurol..

[42] Major E.O., Yousry T.A., Clifford D.B. (2018). Pathogenesis of Progressive Multifocal Leukoencephalopathy and Risks Associated with Treatments for Multiple Sclerosis: A Decade of Lessons Learned. Lancet Neurol..

[43] Hawker K., O’Connor P., Freedman M.S., Calabresi P.A., Antel J., Simon J., Hauser S., Waubant E., Vollmer T., Panitch H. (2009). Rituximab in Patients with Primary Progressive Multiple Sclerosis: Results of a Randomized Double-Blind Placebo-Controlled Multicenter Trial. Ann. Neurol..

[44] Montalban X., Hauser S.L., Kappos L., Arnold D.L., Bar-Or A., Comi G., de Seze J., Giovannoni G., Hartung H.-P., Hemmer B. (2017). Ocrelizumab versus Placebo in Primary Progressive Multiple Sclerosis. N. Engl. J. Med..

[45] Hauser S.L., Bar-Or A., Comi G., Giovannoni G., Hartung H.-P., Hemmer B., Lublin F., Montalban X., Rammohan K.W., Selmaj K. (2017). Ocrelizumab versus Interferon Beta-1a in Relapsing Multiple Sclerosis. N. Engl. J. Med..

[46] Rindi L.V., Zaçe D., Braccialarghe N., Massa B., Barchi V., Iannazzo R., Fato I., De Maria F., Kontogiannis D., Malagnino V. (2024). Drug-Induced Progressive Multifocal Leukoencephalopathy (PML): A Systematic Review and Meta-Analysis. Drug Saf..

[47] Steinman L. (2012). The Discovery of Natalizumab, a Potent Therapeutic for Multiple Sclerosis. J. Cell Biol..

[48] Rudick R.A., Stuart W.H., Calabresi P.A., Confavreux C., Galetta S.L., Radue E.-W., Lublin F.D., Weinstock-Guttman B., Wynn D.R., Lynn F. (2006). Natalizumab plus Interferon Beta-1a for Relapsing Multiple Sclerosis. N. Engl. J. Med..

[49] Polman C.H., O’Connor P.W., Havrdova E., Hutchinson M., Kappos L., Miller D.H., Phillips J.T., Lublin F.D., Giovannoni G., Wajgt A. (2006). A Randomized, Placebo-Controlled Trial of Natalizumab for Relapsing Multiple Sclerosis. N. Engl. J. Med..

[50] Ghezzi A., Moiola L., Pozzilli C., Brescia-Morra V., Gallo P., Grimaldi L.M.E., Filippi M., Comi G. G., MS Study Group-Italian Society of Neurology (2015). Natalizumab in the Pediatric MS Population: Results of the Italian Registry. BMC Neurol..

[51] Margoni M., Rinaldi F., Riccardi A., Franciotta S., Perini P., Gallo P. (2020). No Evidence of Disease Activity Including Cognition (NEDA-3 plus) in Naïve Pediatric Multiple Sclerosis Patients Treated with Natalizumab. J. Neurol..

[52] Gontika M., Skarlis C., Markoglou N., Tzanetakos D., Vakrakou A., Toulas P., Koutsis G., Evangelopoulos M.-E., Pons R., Dardiotis E. (2022). Natalizumab Therapy in Patients with Pediatric-Onset Multiple Sclerosis in Greece: Clinical and Immunological Insights of Time-Long Administration and Future Directions-a Single-Center Retrospective Observational Study. Naunyn-Schmiedeberg’s Arch. Pharmacol..

[53] Sirbu C.A., Ghinescu M.C., Axelerad A.D., Sirbu A.M., Ionita-Radu F. (2021). A New Era for Monoclonal Antibodies with Applications in Neurology (Review). Exp. Ther. Med..

[54] Alvarez E., Steinman L., Fox E.J., Hartung H.-P., Qian P., Wray S., Robertson D., Selmaj K., Wynn D., Mok K. (2024). Improvements in No Evidence of Disease Activity with Ublituximab vs. Teriflunomide in the ULTIMATE Phase 3 Studies in Relapsing Multiple Sclerosis. Front. Neurol..

[55] Smets I., Giovannoni G. (2022). Derisking CD20-Therapies for Long-Term Use. Mult. Scler. Relat. Disord..

[56] DEMİR S., ATMACA M.M., TOGROL R.E. (2019). The First Cure Experience of A Clinic: Approach to The Patient to Start Ocrelizumab. Noro Psikiyatr. Ars..

[57] Wang M., Otto C., Fernández Zapata C., Dehlinger A., Gallaccio G., Diekmann L.-M., Niederschweiberer M., Schindler P., Körtvélyessy P., Kunkel D. (2025). Comprehensive Analysis of B Cell Repopulation in Ocrelizumab-Treated Patients with Multiple Sclerosis by Mass Cytometry and Proteomics. iScience.

[58] Coles A.J., Twyman C.L., Arnold D.L., Cohen J.A., Confavreux C., Fox E.J., Hartung H.-P., Havrdova E., Selmaj K.W., Weiner H.L. (2012). Alemtuzumab for Patients with Relapsing Multiple Sclerosis after Disease-Modifying Therapy: A Randomised Controlled Phase 3 Trial. Lancet.

[59] Holmøy T., von der Lippe H., Leegaard T.M. (2017). Listeria Monocytogenes Infection Associated with Alemtuzumab—A Case for Better Preventive Strategies. BMC Neurol..

[60] Croteau D., Flowers C., Kulick C.G., Brinker A., Kortepeter C.M. (2018). Acute Acalculous Cholecystitis: A New Safety Risk for Patients with MS Treated with Alemtuzumab. Neurology.

[61] Cadavid D., Mellion M., Hupperts R., Edwards K.R., Calabresi P.A., Drulović J., Giovannoni G., Hartung H.-P., Arnold D.L., Fisher E. (2019). Safety and Efficacy of Opicinumab in Patients with Relapsing Multiple Sclerosis (SYNERGY): A Randomised, Placebo-Controlled, Phase 2 Trial. Lancet Neurol..

[62] Eisen A., Greenberg B.M., Bowen J.D., Arnold D.L., Caggiano A.O. (2017). A Double-Blind, Placebo-Controlled, Single Ascending-Dose Study of Remyelinating Antibody rHIgM22 in People with Multiple Sclerosis. Mult. Scler. J. Exp. Transl. Clin..

[63] Giovannoni G., Gold R., Selmaj K., Havrdova E., Montalban X., Radue E.-W., Stefoski D., McNeill M., Amaravadi L., Sweetser M. (2014). Daclizumab High-Yield Process in Relapsing-Remitting Multiple Sclerosis (SELECTION): A Multicentre, Randomised, Double-Blind Extension Trial. Lancet Neurol..

[64] Fox R.J., Bar-Or A., Traboulsee A., Oreja-Guevara C., Giovannoni G., Vermersch P., Syed S., Li Y., Vargas W.S., Turner T.J. (2025). Tolebrutinib in Nonrelapsing Secondary Progressive Multiple Sclerosis. N. Engl. J. Med..

[65] Oh J., Arnold D.L., Cree B.A.C., Ionete C., Kim H.J., Sormani M.P., Syed S., Chen Y., Maxwell C.R., Benoit P. (2025). Tolebrutinib versus Teriflunomide in Relapsing Multiple Sclerosis. N. Engl. J. Med..

[66] Bar-Or A., Dufek M., Budincevic H., Drulovic J., Habek M., Hua L.H., Weber M.S., Thomas P., Napieralski J., Mitzner M.C. (2025). Safety and Efficacy of Fenebrutinib in Relapsing Multiple Sclerosis (FENopta): A Multicentre, Double-Blind, Randomised, Placebo-Controlled, Phase 2 Trial and Open-Label Extension Study. Lancet Neurol..

[67] Hatchwell E., Smith E.B., Jalilzadeh S., Bruno C.D., Taoufik Y., Hendel-Chavez H., Liblau R., Brassat D., Martin-Blondel G., Wiendl H. (2022). Progressive Multifocal Leukoencephalopathy Genetic Risk Variants for Pharmacovigilance of Immunosuppressant Therapies. Front. Neurol..

[68] Skarlis C., Papadopoulos V., Raftopoulou S., Mavragani C.P., Evangelopoulos M.-E. (2024). Association of B-Cell Activating Factor Gene Variants with Serum Anti-JCV Antibody Positivity in Male Patients with Multiple Sclerosis under Natalizumab Treatment: Implications for Progressive Multifocal Leukoencephalopathy Risk Stratification. J. Neurol. Sci..

[69] de la Hera B., Urcelay E., Brassat D., Chan A., Vidal-Jordana A., Salmen A., Villar L.M., Álvarez-Cermeño J.C., Izquierdo G., Fernández O. (2014). Natalizumab-Related Anaphylactoid Reactions in MS Patients Are Associated with HLA Class II Alleles. Neurol. Neuroimmunol. Neuroinflamm..

[70] Chu M.M., Li V., Kalincik T., Lui E., Seet M.-R., Jackson S., Kilpatrick T. (2025). A Single Nucleotide Polymorphism in the MerTK Gene Is Associated with Increased Radiological Disease Activity in Patients with Multiple Sclerosis on Natalizumab Therapy. Mult. Scler. Relat. Disord..

[71] Carlson A.K., Amin M., Cohen J.A. (2024). Drugs Targeting CD20 in Multiple Sclerosis: Pharmacology, Efficacy, Safety, and Tolerability. Drugs.

[72] Bar-Or A., Grove R.A., Austin D.J., Tolson J.M., VanMeter S.A., Lewis E.W., Derosier F.J., Lopez M.C., Kavanagh S.T., Miller A.E. (2018). Subcutaneous Ofatumumab in Patients with Relapsing-Remitting Multiple Sclerosis: The MIRROR Study. Neurology.

[73] Protopapa M., Schraad M., Pape K., Steffen F., Steenken L., Zipp F., Fleischer V., Bittner S. (2025). Recurrent Late-Onset Neutropenia Following Treatment with Different B Cell-Depleting Strategies in Multiple Sclerosis. Med.

[74] Steinman L., Fox E., Hartung H.-P., Alvarez E., Qian P., Wray S., Robertson D., Huang D., Selmaj K., Wynn D. (2022). Ublituximab versus Teriflunomide in Relapsing Multiple Sclerosis. N. Engl. J. Med..

[75] Coles A.J., Cohen J.A., Fox E.J., Giovannoni G., Hartung H.-P., Havrdova E., Schippling S., Selmaj K.W., Traboulsee A., Compston D.A.S. (2017). Alemtuzumab CARE-MS II 5-Year Follow-up: Efficacy and Safety Findings. Neurology.

[76] Havrdova E., Arnold D.L., Cohen J.A., Hartung H.-P., Fox E.J., Giovannoni G., Schippling S., Selmaj K.W., Traboulsee A., Compston D.A.S. (2017). Alemtuzumab CARE-MS I 5-Year Follow-up: Durable Efficacy in the Absence of Continuous MS Therapy. Neurology.

[77] Berger T., Elovaara I., Fredrikson S., McGuigan C., Moiola L., Myhr K.-M., Oreja-Guevara C., Stoliarov I., Zettl U.K. (2017). Alemtuzumab Use in Clinical Practice: Recommendations from European Multiple Sclerosis Experts. CNS Drugs.

[78] Kazakou P., Tzanetakos D., Vakrakou A.G., Tzartos J.S., Evangelopoulos Μ.-E., Anagnostouli M., Stathopoulos P., Kassi G.N., Stefanis L., Kilidireas C. (2023). Thyroid Autoimmunity Following Alemtuzumab Treatment in Multiple Sclerosis Patients: A Prospective Study. Clin. Exp. Med..

[79] Saarela M., Senthil K., Jones J., Tienari P.J., Soilu-Hänninen M., Airas L., Coles A., Saarinen J.T. (2018). Hemophagocytic Lymphohistiocytosis in 2 Patients with Multiple Sclerosis Treated with Alemtuzumab. Neurology.

[80] Ferraro D., Camera V., Vitetta F., Zennaro M., Ciolli L., Nichelli P.F., Sola P. (2018). Acute Coronary Syndrome Associated with Alemtuzumab Infusion in Multiple Sclerosis. Neurology.

[81] Cadavid D., Balcer L., Galetta S., Aktas O., Ziemssen T., Vanopdenbosch L., Frederiksen J., Skeen M., Jaffe G.J., Butzkueven H. (2017). Safety and Efficacy of Opicinumab in Acute Optic Neuritis (RENEW): A Randomised, Placebo-Controlled, Phase 2 Trial. Lancet Neurol..

[82] Krämer J., Wiendl H. (2022). What Have Failed, Interrupted, and Withdrawn Antibody Therapies in Multiple Sclerosis Taught Us?. Neurotherapeutics.

[83] Vermersch P., Granziera C., Mao-Draayer Y., Cutter G., Kalbus O., Staikov I., Dufek M., Saubadu S., Bejuit R., Truffinet P. (2024). Inhibition of CD40L with Frexalimab in Multiple Sclerosis. N. Engl. J. Med..

[84] Fatima T., Mirza A., Fatima F., Karamat R.I., Ahmad B., Naeem S., Shahid I., Akilimali A. (2024). Frexalimab (SAR441344) as a Potential Multiautoimmune Disorder Tackling mAB Targeting the CD40-CD40L Pathway Undergoing Clinical Trials: A Review. Ann. Med. Surg..

[85] Chitnis T., Singhal T., Zurawski J., Saraceno T.J., Gopalakrishnan N., Cain L., LaBarre B., King D., Bergmark R.W., Maxfield A.Z. (2025). Nasal Foralumab Treatment of PIRA Induces Regulatory Immunity, Dampens Microglial Activation and Stabilizes Clinical Progression in Non-Active Secondary Progressive MS. medRxiv.

[86] Siriratnam P., Huda S., Butzkueven H., van der Walt A., Jokubaitis V., Monif M. (2023). A Comprehensive Review of the Advances in Neuromyelitis Optica Spectrum Disorder. Autoimmun. Rev..

[87] Carnero Contentti E., Correale J. (2021). Neuromyelitis Optica Spectrum Disorders: From Pathophysiology to Therapeutic Strategies. J. Neuroinflammation.

[88] Huang T.-L., Wang J.-K., Chang P.-Y., Hsu Y.-R., Lin C.-H., Lin K.-H., Tsai R.-K. (2022). Neuromyelitis Optica Spectrum Disorder: From Basic Research to Clinical Perspectives. Int. J. Mol. Sci..

[89] Shen X. (2023). Research Progress on Pathogenesis and Clinical Treatment of Neuromyelitis Optica Spectrum Disorders (NMOSDs). Clin. Neurol. Neurosurg..

[90] Giglhuber K., Berthele A. (2022). Adverse Events in NMOSD Therapy. Int. J. Mol. Sci..

[91] Rigal J., Pugnet G., Ciron J., Lépine Z., Biotti D. (2020). Off-Label Use of Tocilizumab in Neuromyelitis Optica Spectrum Disorders and MOG-Antibody-Associated Diseases: A Case-Series. Mult. Scler. Relat. Disord..

[92] Ramirez A.D., Dresser L., Abuaf A., Javed A. (2025). Treatment Options for NMOSD in Children: Effectiveness and Safety of Subcutaneous Tocilizumab (P12-8.009). Neurology.

[93] Wang Y., Zhao M., Yao M., Yang Z., Li B., Yin L., Geng X. (2023). Tocilizumab Treatment in Neuromyelitis Optica Spectrum Disorders: Updated Meta-Analysis of Efficacy and Safety. Mult. Scler. Relat. Disord..

[94] FDA Approves Alexion’s Eculizumab for Neuromyelitis Optica Spectrum Disorder. https://www.centerforbiosimilars.com/view/fda-approves-alexions-eculizumab-for-neuromyelitis-optica-spectrum-disorder-.

[95] Food and Drug Administration (2024). ULTOMIRIS® (ravulizumab-cwvz) Injection, for Intravenous Use. https://www.accessdata.fda.gov/drugsatfda_docs/label/2022/761108s023lbl.pdf.

[96] Balaban D.T., Levy M., Borrow R., Anderson M.R. (2024). An Evaluation of Ravulizumab for the Treatment of Neuromyelitis Optica Spectrum Disorder. Expert. Opin. Biol. Ther..

[97] Cree B.A.C., Kim H.J., Weinshenker B.G., Pittock S.J., Wingerchuk D.M., Fujihara K., Paul F., Cutter G.R., Marignier R., Green A.J. (2024). Safety and Efficacy of Inebilizumab for the Treatment of Neuromyelitis Optica Spectrum Disorder: End-of-Study Results from the Open-Label Period of the N-MOmentum Trial. Lancet Neurol..

[98] Cree B.A.C., Bennett J.L., Kim H.J., Weinshenker B.G., Pittock S.J., Wingerchuk D.M., Fujihara K., Paul F., Cutter G.R., Marignier R. (2019). Inebilizumab for the Treatment of Neuromyelitis Optica Spectrum Disorder (N-MOmentum): A Double-Blind, Randomised Placebo-Controlled Phase 2/3 Trial. Lancet.

[99] Ali F., Sharma K., Anjum V., Ali A. (2022). Inebilizumab-Cdon: USFDA Approved for the Treatment of NMOSD (Neuromyelitis Optica Spectrum Disorder). Curr. Drug Discov. Technol..

[100] Stone J.H., Khosroshahi A., Zhang W., Della Torre E., Okazaki K., Tanaka Y., Löhr J.M., Schleinitz N., Dong L., Umehara H. (2025). Inebilizumab for Treatment of IgG4-Related Disease. N. Engl. J. Med..

[101] Pittock S.J., Lennon V.A., McKeon A., Mandrekar J., Weinshenker B.G., Lucchinetti C.F., O’Toole O., Wingerchuk D.M. (2013). Eculizumab in AQP4-IgG-Positive Relapsing Neuromyelitis Optica Spectrum Disorders: An Open-Label Pilot Study. Lancet Neurol..

[102] Clardy S.L., Pittock S.J., Aktas O., Nakahara J., Isobe N., Centonze D., Fam S., Kielhorn A., Yu J.C., Jansen J. (2024). Network Meta-Analysis of Ravulizumab and Alternative Interventions for the Treatment of Neuromyelitis Optica Spectrum Disorder. Neurol. Ther..

[103] Bennett J.L., Aktas O., Rees W.A., Smith M.A., Gunsior M., Yan L., She D., Cimbora D., Pittock S.J., Weinshenker B.G. (2022). Association between B-Cell Depletion and Attack Risk in Neuromyelitis Optica Spectrum Disorder: An Exploratory Analysis from N-MOmentum, a Double-Blind, Randomised, Placebo-Controlled, Multicentre Phase 2/3 Trial. EBioMedicine.

[104] Uplizna® (Inebilizumab-Cdon) Is Now the First and Only Fda-Approved Treatment for Igg4-Related Disease. https://www.amgen.com/newsroom/press-releases/2025/04/uplizna-inebilizumabcdon-is-now-the-first-and-only-fdaapproved-treatment-for-igg4related-disease.

[105] Zhang X.-X., Tian Y., Wang Z.-T., Ma Y.-H., Tan L., Yu J.-T. (2021). The Epidemiology of Alzheimer’s Disease Modifiable Risk Factors and Prevention. J. Prev. Alzheimer’s Dis..

[106] Jucker M., Walker L.C. (2023). Alzheimer’s Disease: From Immunotherapy to Immunoprevention. Cell.

[107] Beshir S.A., Hussain N., Menon V.B., Al Haddad A.H.I., Al Zeer R.A.h., Elnour A.A. (2024). Advancements and Challenges in Antiamyloid Therapy for Alzheimer’s Disease: A Comprehensive Review. Int. J. Alzheimers Dis..

[108] Liu A., Wang T., Yang L., Zhou Y. (2025). The APOE-Microglia Axis in Alzheimer’s Disease: Functional Divergence and Therapeutic Perspectives-A Narrative Review. Brain Sci..

[109] Topalis V., Voros C., Ziaka M. (2025). Targeting Inflammation in Alzheimer’s Disease: Insights into Pathophysiology and Therapeutic Avenues—A Comprehensive Review. J. Geriatr. Psychiatry Neurol..

[110] Haddad H.W., Malone G.W., Comardelle N.J., Degueure A.E., Kaye A.M., Kaye A.D. (2022). Aducanumab, a Novel Anti-Amyloid Monoclonal Antibody, for the Treatment of Alzheimer’s Disease: A Comprehensive Review. Health Psychol. Res..

[111] Brockmann R., Nixon J., Love B.L., Yunusa I. (2023). Impacts of FDA Approval and Medicare Restriction on Antiamyloid Therapies for Alzheimer’s Disease: Patient Outcomes, Healthcare Costs, and Drug Development. Lancet Reg. Health Am..

[112] Dhillon S. (2021). Aducanumab: First Approval. Drugs.

[113] van Dyck C.H., Swanson C.J., Aisen P., Bateman R.J., Chen C., Gee M., Kanekiyo M., Li D., Reyderman L., Cohen S. (2023). Lecanemab in Early Alzheimer’s Disease. N. Engl. J. Med..

[114] Hoy S.M. (2023). Lecanemab: First Approval. Drugs.

[115] Babar Z., Babar A. (2025). FDA’s New Approval; Kisunla (Donanemab-Azbt): A Major Breakthrough in Alzheimer’s Care. J. Pak. Med. Assoc..

[116] Siebrand C.J., Bergo N.J., Lee S., Andersen J.K., Walton C.C. (2025). Chimeric Antigen Receptors Discriminate between Tau and Distinct Amyloid-Beta Species. J. Transl. Med..

[117] Walsh S., Howard R., Richard E., Milne R., Brayne C. (2025). Interpreting the Evidence on Gantenerumab for Dominantly Inherited Alzheimer’s Disease. Lancet Neurol..

[118] McCullough A., Chen C.D., Gordon B.A., Joseph-Mathurin N., Jack C.R., Koeppe R., Hornbeck R., Koudelis D., McKay N.S., Hobbs D.A. (2025). Regional Effects of Gantenerumab on Neuroimaging Biomarkers in the DIAN-TU-001 Trial. Alzheimers Dement..

[119] Ostrowitzki S., Bittner T., Sink K.M., Mackey H., Rabe C., Honig L.S., Cassetta E., Woodward M., Boada M., van Dyck C.H. (2022). Evaluating the Safety and Efficacy of Crenezumab vs Placebo in Adults with Early Alzheimer Disease: Two Phase 3 Randomized Placebo-Controlled Trials. JAMA Neurol..

[120] Grimm H.P., Schumacher V., Schäfer M., Imhof-Jung S., Freskgård P.-O., Brady K., Hofmann C., Rüger P., Schlothauer T., Göpfert U. (2023). Delivery of the Brainshuttle^TM^ Amyloid-Beta Antibody Fusion Trontinemab to Non-Human Primate Brain and Projected Efficacious Dose Regimens in Humans. Mabs.

[121] Muliaditan M., van Steeg T.J., Avery L.B., Sun W., Hammond T.R., Hijdra D., Choi S.-L., Pillai N., Leksa N.C., Mavroudis P.D. (2025). Translational Minimal Physiologically Based Pharmacokinetic Model for Transferrin Receptor-Mediated Brain Delivery of Antibodies. MAbs.

[122] Fleisher A.S., Munsie L.M., Perahia D.G.S., Andersen S.W., Higgins I.A., Hauck P.M., Lo A.C., Sims J.R., Brys M., Mintun M. (2024). Assessment of Efficacy and Safety of Zagotenemab: Results From PERISCOPE-ALZ, a Phase 2 Study in Early Symptomatic Alzheimer Disease. Neurology.

[123] Dam T., Boxer A.L., Golbe L.I., Höglinger G.U., Morris H.R., Litvan I., Lang A.E., Corvol J.-C., Aiba I., Grundman M. (2021). Safety and Efficacy of Anti-Tau Monoclonal Antibody Gosuranemab in Progressive Supranuclear Palsy: A Phase 2, Randomized, Placebo-Controlled Trial. Nat. Med..

[124] Teng E., Manser P.T., Pickthorn K., Brunstein F., Blendstrup M., Sanabria Bohorquez S., Wildsmith K.R., Toth B., Dolton M., Ramakrishnan V. (2022). Safety and Efficacy of Semorinemab in Individuals with Prodromal to Mild Alzheimer Disease: A Randomized Clinical Trial. JAMA Neurol..

[125] Monteiro C., Toth B., Brunstein F., Bobbala A., Datta S., Ceniceros R., Sanabria Bohorquez S.M., Anania V.G., Wildsmith K.R., Schauer S.P. (2023). Randomized Phase II Study of the Safety and Efficacy of Semorinemab in Participants with Mild-to-Moderate Alzheimer Disease: Lauriet. Neurology.

[126] Florian H., Wang D., Arnold S.E., Boada M., Guo Q., Jin Z., Zheng H., Fisseha N., Kalluri H.V., Rendenbach-Mueller B. (2023). Tilavonemab in Early Alzheimer’s Disease: Results from a Phase 2, Randomized, Double-Blind Study. Brain.

[127] Rashad A., Rasool A., Shaheryar M., Sarfraz A., Sarfraz Z., Robles-Velasco K., Cherrez-Ojeda I. (2022). Donanemab for Alzheimer’s Disease: A Systematic Review of Clinical Trials. Healthcare.

[128] Nguyen H.V., Mital S., Knopman D.S., Alexander G.C. (2024). Cost-Effectiveness of Lecanemab for Individuals with Early-Stage Alzheimer Disease. Neurology.

[129] Willis B.A., Lo A.C., Dage J.L., Shcherbinin S., Chinchen L., Andersen S.W., LaBell E.S., Perahia D.G.S., Hauck P.M., Lowe S.L. (2023). Safety, Tolerability, and Pharmacokinetics of Zagotenemab in Participants with Symptomatic Alzheimer’s Disease: A Phase I Clinical Trial. J. Alzheimers Dis. Rep..

[130] Shulman M., Kong J., O’Gorman J., Ratti E., Rajagovindan R., Viollet L., Huang E., Sharma S., Racine A.M., Czerkowicz J. (2023). TANGO: A Placebo-Controlled Randomized Phase 2 Study of Efficacy and Safety of the Anti-Tau Monoclonal Antibody Gosuranemab in Early Alzheimer’s Disease. Nat. Aging.

[131] Ben-Shlomo Y., Darweesh S., Llibre-Guerra J., Marras C., San Luciano M., Tanner C. (2024). The Epidemiology of Parkinson’s Disease. Lancet.

[132] Morris H.R., Spillantini M.G., Sue C.M., Williams-Gray C.H. (2024). The Pathogenesis of Parkinson’s Disease. Lancet.

[133] MacMahon Copas A.N., McComish S.F., Fletcher J.M., Caldwell M.A. (2021). The Pathogenesis of Parkinson’s Disease: A Complex Interplay Between Astrocytes, Microglia, and T Lymphocytes?. Front. Neurol..

[134] Funayama M., Nishioka K., Li Y., Hattori N. (2023). Molecular Genetics of Parkinson’s Disease: Contributions and Global Trends. J. Hum. Genet..

[135] Lang A.E., Siderowf A.D., Macklin E.A., Poewe W., Brooks D.J., Fernandez H.H., Rascol O., Giladi N., Stocchi F., Tanner C.M. (2022). Trial of Cinpanemab in Early Parkinson’s Disease. N. Engl. J. Med..

[136] Hutchison R.M., Fraser K., Yang M., Fox T., Hirschhorn E., Njingti E., Scott D., Bedell B.J., Kistner K.M., Cedarbaum J.M. (2024). Cinpanemab in Early Parkinson Disease: Evaluation of Biomarker Results from the Phase 2 SPARK Clinical Trial. Neurology.

[137] Games D., Valera E., Spencer B., Rockenstein E., Mante M., Adame A., Patrick C., Ubhi K., Nuber S., Sacayon P. (2014). Reducing C-Terminal-Truncated Alpha-Synuclein by Immunotherapy Attenuates Neurodegeneration and Propagation in Parkinson’s Disease-like Models. J. Neurosci..

[138] Pagano G., Taylor K.I., Anzures-Cabrera J., Marchesi M., Simuni T., Marek K., Postuma R.B., Pavese N., Stocchi F., Azulay J.-P. (2022). Trial of Prasinezumab in Early-Stage Parkinson’s Disease. N. Engl. J. Med..

[139] Zheng X., Wang M., He Q., Chen S., Simayi D., Ma X., Zhao J., Sun X., Yang P., Mao Q. (2024). Production and Characterization of Novel Monoclonal Antibodies against Pathological Human TDP-43 Proteins. J. Neuropathol. Exp. Neurol..

[140] van den Bos M.A.J., Geevasinga N., Higashihara M., Menon P., Vucic S. (2019). Pathophysiology and Diagnosis of ALS: Insights from Advances in Neurophysiological Techniques. Int. J. Mol. Sci..

[141] Longinetti E., Regodón Wallin A., Samuelsson K., Press R., Zachau A., Ronnevi L.-O., Kierkegaard M., Andersen P.M., Hillert J., Fang F. (2018). The Swedish Motor Neuron Disease Quality Registry. Amyotroph. Lateral Scler. Front. Degener..

[142] Jun K.Y., Park J., Oh K.-W., Kim E.M., Bae J.S., Kim I., Kim S.H. (2019). Epidemiology of ALS in Korea Using Nationwide Big Data. J. Neurol. Neurosurg. Psychiatry.

[143] Leighton D.J., Newton J., Stephenson L.J., Colville S., Davenport R., Gorrie G., Morrison I., Swingler R., Chandran S., Pal S. (2019). Changing Epidemiology of Motor Neurone Disease in Scotland. J. Neurol..

[144] Eisen A., Vucic S., Mitsumoto H. (2024). History of ALS and the Competing Theories on Pathogenesis: IFCN Handbook Chapter. Clin. Neurophysiol. Pract..

[145] Zhou W., Xu R. (2023). Current Insights in the Molecular Genetic Pathogenesis of Amyotrophic Lateral Sclerosis. Front. Neurosci..

[146] Kiani L. (2024). ALS Pathogenesis Linked to Actin Barrier Collapse. Nat. Rev. Neurol..

[147] McGuigan A., Blair H.A. (2025). Tofersen: A Review in Amyotrophic Lateral Sclerosis Associated with SOD1 Mutations. CNS Drugs.

[148] Castellanos Otero P., Todd T.W., Shao W., Jones C.J., Huang K., Daughrity L.M., Yue M., Sheth U., Gendron T.F., Prudencio M. (2024). Generation and Characterization of Monoclonal Antibodies against Pathologically Phosphorylated TDP-43. PLoS ONE.

